# Are Cancer Stem Cells a Suitable Target for Breast Cancer Immunotherapy?

**DOI:** 10.3389/fonc.2022.877384

**Published:** 2022-04-21

**Authors:** Roberto Ruiu, Antonino Di Lorenzo, Federica Cavallo, Laura Conti

**Affiliations:** Molecular Biotechnology Center, Department of Molecular Biotechnology and Health Sciences, University of Turin, Turin, Italy

**Keywords:** breast cancer, cancer stem cells, immunotherapy, CAR-T, vaccination, monoclonal antibody, clinical trial

## Abstract

There is substantial evidence to suggest that complete tumor eradication relies on the effective elimination of cancer stem cells (CSCs). CSCs have been widely described as mediators of resistance to conventional therapies, including chemo- and radiotherapy, as well as of tumor metastasization and relapse in different tumor types, including breast cancer. However, the resistant phenotype of CSCs makes their targeting a tough task, and immunotherapy may therefore be an interesting option. Nevertheless, although immunotherapeutic approaches to cancer treatment have generated great enthusiasm due to recent success in clinics, breast cancer treatment mostly relies on standard approaches. In this context, we review the existing literature on the immunological properties of breast CSC and immunotherapeutic approaches to them. We will thus attempt to clarify whether there is room for the immunotargeting of breast CSCs in the current landscape of breast cancer therapies. Finally, we will provide our opinion on the CSC-targeting immunotherapeutic strategies that could prospectively be attempted.

## Introduction

Breast cancer (BC) is the most common cancer in women (1), and is still the first cause of cancer-associated mortality in women in several countries due to the development of therapy resistance and subsequent local or distant relapses (2). High inter-tumor and intra-tumor heterogeneity, leading to cell populations with different sensitivities to therapy (3), is a major challenge in BC treatment. The heterogeneity observed during BC progression can be attributed to both genetic and environmental factors, and the presence of Cancer Stem Cells (CSCs) (4, 5). CSCs are a small population of cancer cells with stem-like features that can be functionally defined by their capability to self-renew, differentiate, give rise to all the cell lineages that compose the tumor bulk and seed new tumors (6, 7). CSCs are endowed with plasticity and can generate cells with different functional, phenotypic and metabolic features, inducing tumor heterogeneity. The breast CSC (BCSC) niche contains various non-malignant cells that communicate with CSCs through direct contact and soluble factors to promote self-renewal, radio- and chemotherapy resistance and metastasis generation (8–10). BCSCs were identified (11) as a rare population of cancer cells of a CD44^+^CD24^−/low^Lin^−^ phenotype that possessed higher tumor initiation capacity than BC cells of a different phenotype. Although the presence of BCSCs has since been demonstrated by several research groups, many biological features, including their phenotypic markers and ontology, are still controversial (12). CSCs can originate from a normal adult stem or progenitor cell undergoing neoplastic transformation, non-stem cells that gain self-renewal capabilities upon transformation, and from the de-differentiation of cancer cells without further genomic alterations (13–16). BCSCs are plastic and dynamic and can acquire different features following interactions with the tumor microenvironment and the BCSC niche. Two distinct states of BCSCs have been identified: mesenchymal-like BCSCs, characterized by the CD44^+^CD24^−/low^ phenotype; and epithelial-like BCSCs, which express high levels of the aldehyde dehydrogenase (ALDH)1 enzyme (17). Mesenchymal BCSCs are quiescent, highly migratory, localized in hypoxic areas and at the invasive tumor front, and principally responsible for metastatic dissemination. Conversely, epithelial BCSCs are located in the inner tumor region and are highly proliferative and chemoresistant (18). These two phenotypes can dynamically convert one into the other.

The presence of high CSC levels in a tumor is associated with poor prognosis (19), and CSCs can induce cancer relapse (20, 21), as demonstrated by an enrichment in the CSC-related transcriptional signature observed in the tumor tissue remaining after endocrine therapy and chemotherapy in BC patients (22). Therefore, CSCs are an ideal therapeutic target as their eradication would eliminate the source of tumor progression, heterogeneity and relapse. However, BCSCs activate molecular pathways that render them resistant to current therapies, such as the increased functionality of DNA-repair mechanisms, the overexpression of detoxifying enzymes and efflux pumps, enhanced anti-oxidant capabilities and resistance to apoptosis and other cell-death forms (4, 23). Overall, these features make BCSCs resistant to conventional anti-cancer treatments. The emergence of immunotherapy as a valuable anti-cancer strategy potentially makes it an effective approach to targeting BCSCs, although many open questions remain (15). We herein summarize current knowledge on the interplay between BCSCs and the immune system, review the immunotargeting approaches developed for BCSCs in preclinical and clinical settings (listed in **Table 1**), and discuss the influence that the presence of BCSCs may exert on the immunotherapy outcome.

**Table 1:** Clinical trials on breast cancer stem cell immunotherapy.

Trial	Tumor type	Compound or type of intervention	Description	Phase	Status	Results
NCT01782274	Brain metastases of breast cancer	Dendritic vaccine, allogeneic hematopoietic stem cells, cytotoxic lymphocytes	Driving acute lethal neuroncological processes towords chronic and non-lethal by the control of tumor cells (TCs) quantity and targeted regulation of effector functions of cancer stem cells (CSCs)	II	Completed	Not posted
		Dendritic vaccine, autologous hematopoietic stem cells, cytotoxic lymphocytes		III		
NCT02063893	Breast cancer	CSC-loaded dendritic cell vaccines (ex vivo study)	B and T lymphocytes priming by CSC-loaded dendritic cells		Completed	Not posted
NCT02157051	HER2 negative, stage III-IV breast cancer	Multiantigen DNA vaccination	Assessing side effects and best dose of multiantigen DNA plasmid-based vaccination	I	Recruiting	
NCT04430595	Breast cancer	CAR-T cells	Assessing the feasibility, safety and efficacy of multiple 4th generation CAR-T cells targeting Her2, GD2, and CD44v6 surface antigen in breast cancer.	I-II	Recruiting	
NCT04427449						
NCT02915445	Malignant neoplasm of nasopharynx TNM staging distant metastasis (M), Breast cancer recurrent	CAR-T cells	Assessing the efficacy of CAR-T cells recognizing EpCAM	I	Recruiting	
NCT02541370	Liver Cancer, Pancreatic Cancer, Brain Tumor, Breast Cancer, Ovarian Tumor, Colorectal Cancer, Acute Myeloid and Lymphoid Leukemias	CAR-T cells	Assessing the safety and feasibility of autologous or donor-derived T cells engineered to express an anti-CD133 CAR	I-II	Completed	Published, not on breast cancer
NCT03319459	HER2 Positive Gastric Cancer, Colorectal Cancer, Head and Neck Squamous Cell Carcinoma, EGFR Positive Solid Tumor, Advanced Solid Tumors, HER2-positive Breast Cancer, Hepatocellular Carcinoma, Non Small Cell Lung Cancer, Renal Cell Carcinoma, Pancreatic Cancer, Melanoma	FATE-NK100	Donor-derived NK cells with enhanced anti-tumor activity	I	Completed	Not posted
		FATE-NK100 + Trastuzumab	FATE-NK100 + HER2/neu targeting mAB			
		FATE-NK100 + Cetuximab	FATE-NK100 + EGFR-targeting mAB			
NCT02776917	Breast cancer	Cirmtuzumab + Paclitaxel	Assessing the safety and side effects of ROR1-targeting mAB combined with Paclitaxel	I	Active, not recruiting	
NCT02254005	Breast cancer	Bivatuzumab Mertansine	Assessing maximum tolerated dose (MTD), safety, pharmacokinetics, efficacy of CD44v6-targeting mAB combined with the mertansine	I	Completed	Not posted
NCT02254031	Breast cancer	Bivatuzumab Mertansine	Assessing maximum tolerated dose (MTD), safety, pharmacokinetics, efficacy of CD44v6-targeting mAB combined with the mertansine	I	Terminated	Not posted
NCT05076591	HER2 positive advanced solid tumor, advanced breast cancer, advanced gastric cancer	IMM2902	Evaluating the safety, efficacy, and pharmacokinetics (PK) of IMM2902, a HER2/SIRPα bispecific mAb-Trap antibody-receptor fusion protein, in patients with HER2-expressing advanced solid tumor	I	Not yet recruiting	
NCT03135171	Metastatic HER2 positive breast cancer	Trastuzumab + Pertuzumab + Tocilizumab	Determining the safety, tolerability and recommended Phase 2 dose of anti-IL-6R mAb tocilizumab given with trastuzumab and pertuzumab	I	Completed	Not posted
NCT03424005	Triple negative breast cancer	Capecitabine, Atezolizumab, Ipatasertib, SGN-LIV1A, Bevacizumab, Chemotherapy (Gemcitabine + Carboplatin or Eribulin), Selicrelumab, Tocilizumab, Nab-Paclitaxel, Sacituzumab Govitecan	Evaluating the efficacy and safety of multiple immunotherapy-based treatment combinations	I-II	Recruiting	
NCT02066532	Metastatic Breast Cancer, Breast Carcinoma, HER-2 Positive Breast Cancer	Ruxolitinib	Assessing the safety and efficacy of Ruxolitinib (drug used for myelofibrosis treatment) as anti-cancer therapy in combination with Trastuzumab	I	Completed	Not posted
		Trastuzumab		II		
NCT02041429	Recurrent Breast Cancer, Metastatic Breast Cancer	Ruxolitinib	Evaluating a combination of the drugs paclitaxel and ruxolitinib as a possible treatment for inflammatory breast cancer	I	Completed	Ref (24)
		Paclitaxel		II		
NCT02876302	Inflammatory Breast Cancer (IBC)	Ruxolitinib, Paclitaxel, Doxorubicin, Cyclophosphamide	Studying Ruxolitinib as possible treatment for Inflammatory Breast Cancer (IBC) in combination with other chemotherapeutic agents	II	Active, not recruiting	Not posted
NCT02120417	Breast Cancer	Ruxolitinib, Capecitabine, Placebo	Comparing the overall survival of women with advanced or metastatic HER2-negative breast cancer who received treatment with capecitabine in combination with ruxolitinib versus those who received treatment with capecitabine alone	II	Terminated	Posted, not published
NCT01562873	Breast cancer	Ruxolitinib	Testing the effects of ruxolitinib in patients with breast cancer	II	Terminated	Posted, not published
NCT01594216	Estrogen-receptor Positive Invasive Metastatic Breast Cancer	Ruxolitinib, Exemestane	Determining the preliminary safety and efficacy of Ruxolitinib in combination with Exemestane (anti-tumoral drug)	II	Completed	Not posted
NCT03012230	Stage IV Breast Cancer AJCC v6 and v7, Triple-Negative Breast Carcinoma, Bone metastases of breast cancer	Ruxolitinib Phosphate, Pembrolizumab	Studying the side effects and best dose of ruxolitinib phosphate when given together with pembrolizumab	I	Recruiting	Not posted
NCT02370238	Metastatic Breast Cancer	Paclitaxel, Reparixin	Assessing the safety and efficacy of paclitaxel in combination with reparixin	II	Completed	Posted, not published
NCT02001974	Metastatic Breast Cancer	Paclitaxel, Reparixin	Assessing the safety of paclitaxel in combination with reparexin and their efficacy in the targeting of cancer stem cells, circulating tumor cells and metastases	I	Completed	Posted, not published
NCT01861054	Breast Cancer	Reparixin	Assessing the efficacy of Reparixin as single agent in the period between diagnosis and surgery	II	Terminated	Posted, not published

## Immunological Properties of BCSCs

It has been reported that BCSCs employ several mechanisms to evade the immune response. Firstly, they possess defective antigen-presentation machinery, which is the result of the downregulation of major histocompatibility complex class I (MHC-I) molecules and of molecules involved in the peptide-loading process. This protects them from recognition by CD8+ T cells (25). Nevertheless, the absence of MHC-I may render BCSCs a target for Natural Killer (NK) cells (25). Indeed, the upregulation of NK ligands means that NK cells preferentially kill BCSCs, whether CD44^+^/CD24^-^ (26) or ALDH1^bright^ (27), compared to differentiated cells. Moreover, radiotherapy increases the expression of stress ligands on surviving CSCs, causing increased susceptibility to NK killing (28). However, others have reported BCSCs having reduced susceptibility to NK-mediated killing, due to the downregulation of NK-activating NKG2D ligands, meaning they selectively escape from NK-mediated killing and trastuzumab-induced antibody-dependent-cell-cytotoxicity (ADCC) (29–31). Moreover, BCSCs overexpress many immunosuppressive molecules, including several immune checkpoint (IC) ligands that weaken the activity of NK and T cells. Indeed, BCSCs express high levels of Programmed death-ligand (PD-L)1 and sometimes PD-L2 (32, 33), which, besides inhibiting NK and effector T-cell function by binding to PD1, also directly promote stemness by inducing the AKT-dependent expression of NANOG, Octamer-binding transcription factor (OCT)-4A and B lymphoma Mo-MLV insertion region 1 homolog (BMI1) (34). BCSCs also overexpress CD276 and CD155, two IC ligands that can inhibit T and NK cell killing (35), and CD200 (36), which induces a switch from an antitumor T helper (Th)1 to an immunosuppressive Th2 phenotype (37). Similarly, CD47 is overexpressed on BCSCs and, besides inhibiting their phagocytosis by macrophages *via* binding to the signal regulatory protein alpha (SIRPα) receptor and acting as a “Don’t eat me” signal, it directly protects CSCs from apoptosis (38).

Additionally, CSCs secrete immunosuppressive cytokines and other molecules that convert immune cells into tumor allies (4, 39), such as transforming growth factor (TGF)-β, interleukin (IL)-6, IL-8 and vascular endothelial growth factor (VEGF) (40, 41). We have previously demonstrated that BCSCs produce these cytokines as a consequence of an autocrine loop mediated by the overexpression of Toll-like receptor 2 and its engagement by high-mobility group box (HMGB)1 (42, 43). TGF-β plays a complex role in BC progression as it induces epithelial-to-mesenchymal-transition (EMT), and thus acts as a source of stem-like cells and promotes metastases. Along with VEGF, it promotes angiogenesis and induces immunosuppression by favoring T regulatory cells (Tregs) and myeloid-derived suppressor cell (MDSC) infiltration into tumors (44). IL-6 promotes the production of immunosuppressive cytokines IL-10 and IL-21 by Tregs, potentiating their activity (45), and reduces the expression of MHC-II and costimulatory molecules on dendritic cells (DC), thus impairing their ability to activate anti-cancer T cells (46). Whether secreted by tumor or immune cells, IL-6 further sustains BC’s stem-like qualities and progression by stimulating the Signal Transducer and Activator of Transcription (STAT)3-dependent expression of genes involved in stemness, tumorigenesis, migration and metastases (44, 47, 48). The inhibition of MDSC-derived IL-6 extends survival in BC mouse models (49). IL-8 is another cytokine that increases CSC frequency, and its receptor chemokine C-X-C motif receptor (CXCR)1 is more highly expressed in ALDH1^+^ than in ALDH1^–^ BC cells (50). Furthermore, chemotherapy-induced cytotoxicity may increase local IL-8 levels, thus boosting CSC proportions. Indeed, high levels of IL-6 and IL-8 in advanced BC patient sera have been correlated with metastasis development and low therapeutic efficacy (51).

## Preclinical and Clinical Immunotherapeutic Strategies to Target BCSCs

### Vaccines

DC vaccines are common experimental vaccines in BC treatment, despite minimal evidence of clinical activity as a single agent (52). Preclinical studies have demonstrated that DC, pulsed with human BCSC-lysates, prolonged the survival of tumor-bearing mice with humanized immune systems, although no comparison with non-CSC lysates was performed (53). While DC-based vaccines specifically directed against BCSCs are under evaluation in clinics, trial outcome is currently unknown (NCT01782274, NCT02063893).

Besides DC-based vaccines, other vaccine formulations and antigen sources have been exploited to target BCSCs in preclinical studies. Vaccines consisting of irradiated induced pluripotent stem cells (iPSC) resulted in reductions in tumor growth due to the shared antigens in iPSCs and CSCs (54, 55). Our group has tested several vaccine platforms (DNA, oncolytic virus, virus-like particles) to target the CSC-antigen xCT (56–60). These, together with DNA vaccines that target Cripto-1 (61) and peptide-based vaccines targeting Hypoxia-Inducible Factor (HIF)-1α (62), have all demonstrated their efficacy against BCSCs. One DNA-vector-based vaccine, designed to target defined BCSC antigens (CD105/Yb-1/SOX2/CDH3/MDM2), is currently on trial (NCT02157051).

### Adoptive Cell Therapy and CAR-T Cells

Besides vaccines, CSC-primed T cells and chimeric antigen receptor (CAR)-T cells have been developed to generate CSC-targeted immune responses. CSC-primed T cells are generated by stimulating donor CD8+ T cells *in vitro* with autologous DC pulsed with CSC-derived peptides, and are then infused back into the host. For instance, the *in-vitro* stimulation of human CD8+ T cells with autologous DC, pulsed with an ALDH1 peptide, triggered ALDH1-specific CD8+ T cells that restrained BC metastasis and improved survival when transferred into tumor-bearing immunodeficient mice (63).

However, the frequent downregulation of MHC-I molecules and antigen presentation in CSCs (37) can negatively affect the perspective use of T-cell adoptive transfer against CSCs. This hurdle can be overcome by CAR-T cells. These demonstrated activity in patients, leading to the FDA approval of CAR-T cells targeting CD19 in non-Hodgkin lymphomas and pediatric acute lymphoblastic leukemia (64). In preclinical models of BC, CAR-T cells targeting the BCSC-associated antigen GD2 efficiently impaired tumor growth and prevented metastasis formation, despite GD2 only being expressed on a small minority of cells (65). Similar observations were made in CAR-T that target the CSC-associated antigen Tumor endothelial marker 8 (66). CAR-T cells that target BCSC markers, in particular Human Epidermal Growth Factor Receptor (HER)2, GD2 and CD44v6 (NCT04430595), CD44v6 (NCT04427449), Epithelial Cell Adhesion Molecule (EpCam, NCT02915445) and CD133 (NCT02541370), are currently undergoing clinical studies involving BC patients.

The administration of γδ T lymphocytes, which are non-MHC-restricted and bear an invariant γδ TCR, is another approach that can bypass MHC-I downregulation. Clinical testing in BC patients revealed that the adoptive transfer of γδ T cells is safe and feasible, although proof of efficacy is lacking (67). Moving on, the innate effector properties of NK cells make them suitable candidates for immunotherapy. However, the controversial susceptibility of CSCs to NK cell killing, discussed in Section 2, means that there is no consensus on the efficacy of using NK to target BCSCs. Clinical trials using NK cell infusion to treat advanced solid tumors, including BC (NCT03319459), have been completed, although no results have yet been reported.

### Monoclonal Antibodies

Monoclonal antibodies (mAb), including mAbs with direct inhibitory effects and the capacity to elicit ADCC in cancer cells, mAb-drug conjugates that deliver cytotoxic agents and mAb that target ICs, have proven to be a feasible strategy for cancer treatment.

Antibody-based therapies targeting CSC-maintaining pathways, such as the Wingless-related integration site (Wnt) pathway, have reached clinics. Vantictumab is a human mAb that suppresses the induction of the canonical Wnt signaling pathway *via* binding to the frizzled (Fzd) 1, 2, 5, 7 and 8 receptors. This agent reduced the frequency of CSCs and tumor growth in patient-derived xenografts (PDX) models of various tumor types, including BC, and exhibited synergistic activity with standard-of-care chemotherapy (68). In a phase Ib study of vantictumab with paclitaxel involving patients with metastatic HER2- BC, 33% of patients had a partial response and 29% had stable disease (69). Cirmtuzumab is a mAb that targets Receptor-tyrosine-kinase-like-orphan-receptor1, a receptor for Wnt5 found on BCSCs. Treating BC PDX mice with cirmtuzumab repressed the expression of genes associated with stemness and impaired cancer cell capacity to metastasize and regraft. Combination with paclitaxel further improved single-agent efficacy (70). A phase I study of cirmtuzumab plus paclitaxel in BC patients is ongoing (NCT02776917). mAbs against the CSC marker CD44 have been tested in preclinical models of BC (71), and the mAb-drug conjugate bivatuzumab mertansine, targeting CD44v6, has been tested in BC patients (NCT02254005, NCT02254031). However, no recent news is available on the development of this agent.

mAbs that target ICs, so-called IC inhibitors (ICIs), are currently used to treat different cancer types, alone and in combination with other treatments. The anti-PD-L1 atezolizumab has been approved for use in combination with nab-paclitaxel to treat metastatic Triple Negative Breast Cancer (TNBC) (72). As the expression levels of PD-L1 are higher in BCSCs than in non-CSCs (73–75), and as it has a role in promoting both their immune-evading phenotype and stemness (34), targeting PD-L1 may decrease the BCSC pool and improve the efficacy of combined treatments. For example, anti-PD-L1 treatment, in combination with an anti-ALDH1 vaccine, exerted potent antitumor efficacy and prolonged survival in multiple cancer murine models, including BC (76). Anti-PD-L1 treatment might also potentiate adoptive cell therapy. Indeed, increased levels of PD-L1 in CSCs render the γδ T-cells hypo-responsive. CSC sensitivity to γδ-mediated killing can thus be restored *via* PD-1 blockade (77, 78).

Another promising approach for targeting CSCs is to enhance phagocytosis using anti-CD47 mAbs (38). The anti-CD47 antibody Hu5F9-G4 displayed a manageable safety profile in a phase I trial on patients suffering from advanced solid tumors, including BC (79). To avoid the phagocytosis of normal cells, as CD47 is ubiquitous, an HER2 mAb was fused with SIRPα to generate a bispecific mAb-Trap antibody-receptor fusion protein that will be tested in patients with HER2-expressing advanced solid tumors (NCT05076591).

mAbs can be used to revert the immunosuppressive tumor microenvironment and hinder CSC self-renewal by targeting cytokines such as IL-6 and IL-8 (48, 80, 81). Tocilizumab, a mAb that targets the IL-6 receptor, suppresses the stem-like properties of BCSCs and their metastatic ability, potentiating the cytotoxicity of chemotherapy in TNBC (82). The FDA has approved this drug for rheumatoid arthritis and it is now under study for BC treatment (NCT03135171, NCT03424005).

### Small Molecules

Besides mAb, small molecules have also been used to target the CSC/immune-microenvironment interaction and have reached the clinic; for example, ruxolitinib inhibits the IL-6/JAK/STAT pathway (NCT02066532, NCT02041429, NCT02876302, NCT02120417, NCT01562873, NCT01594216, NCT03012230) with evidence of clinical activity (24), while reparixin targets the IL-8 receptor CXCR1, selectively depletes CSCs *in vitro* and reduces tumor growth and metastasization *in vivo* (81). Reparixin has moved to clinical trials (83), giving promising results in terms of safety (84) and efficacy in reducing BCSC frequency (85, 86). Other trials involving reparixin to target CSCs have been recently completed (NCT02370238, NCT02001974, NCT01861054).

## Discussion

Although many immunological approaches for targeting CSCs have given promising results in clinical trials, no CSC-targeting immunotherapy has yet been approved. Moreover, the CSC concept has rarely been implemented in clinical practice, possibly because of the elusive phenotype and exiguous number of CSCs.

The CSC theory is often seen as opposing the clonal evolution theory (87), which is still the mainstream explanation for observed intra-tumor heterogeneity. There may therefore be less interest in assessing the clinical efficacy of CSC-targeted therapies than in those targeting altered pathways and molecules expressed by the tumor bulk. However, the high expression of putative CSC markers is consistently associated with poorer overall survival in BC patients (88), and the presence of residual ALDH1^+^ cells after neoadjuvant therapy is associated with increased metastasization risk (89), indicating that targeting CSCs would have a beneficial impact on patient life expectancy. Nevertheless, evaluating the efficacy of any therapy on the CSC subset is difficult as they are only a minor fraction of cells and are hard to detect with routine tumor-biopsy methods, i.e., IHC, IF, FISH. Currently, no standardized approaches to assess and quantify CSCs within tumor biopsies exist. Moreover, commonly employed clinical endpoints, such as tumor shrinkage, would not reveal any therapeutic effect on CSCs, which may remain as a quiescent residual population before reactivation and relapse.

The lack of a unique marker associated to stemness that can be exploited for CSC detection is another hurdle. Indeed, CSC markers may vary according to the mammary cancer subtype, and different BCSC populations, with different properties (and markers), may even exist within the same tumor. As mentioned, mesenchymal-like and epithelial-like BCSCs co-exist in different proportions according to BC subtype (89). Furthermore, BCSC phenotype is not stable, and different BCSC populations can switch after endogenous (i.e., microenvironmental) and exogenous (i.e., therapeutic) pressure. Similarly, a CSC can differentiate towards a bulk cell, which can conversely de-differentiate to CSC status. CSCs are therefore a moving target, and difficulties in their ontology consequently bring difficulties in their treatment.

The switch between one CSC phenotype and another is accompanied by a switch in associated markers and, thus, targetable antigens. Effective therapy should therefore not rely on one target antigen, but include a set of CSC-associated antigens. However, not all the antigens for the many CSC phenotypes are known, thus limiting the window of intervention for antigen-targeted strategies. CSC-derived lysates have therefore been efficiently used as an (unselected) antigen source for DC loading, in preclinical studies involving squamous cell carcinoma and melanoma (90). CSC-loaded DCs were more potent in inducing an anti-tumor immune response than unselected cancer cells, suggesting that CSCs express a different and underrepresented set of antigens, and that therapeutic efficacy can only be achieved by eliminating CSCs. This proof-of-concept is extremely relevant in the context of the reported difficulty in treating BC with immunotherapy. BC carries a lower load of mutations, and thus of neoantigens, than melanoma and lung cancer. This has a negative impact on the generation of spontaneous anti-tumor T-cell responses and has been associated to the poor efficacy of ICIs in BC (91). Nevertheless, preclinical proof that BCSC-associated antigens represent an activatable vulnerability in BC treatment may pave the way for the use of vaccine platforms in which BCSC-antigen-specific T-cell responses can be triggered, thus improving ICI efficacy.

**Figure 1:**
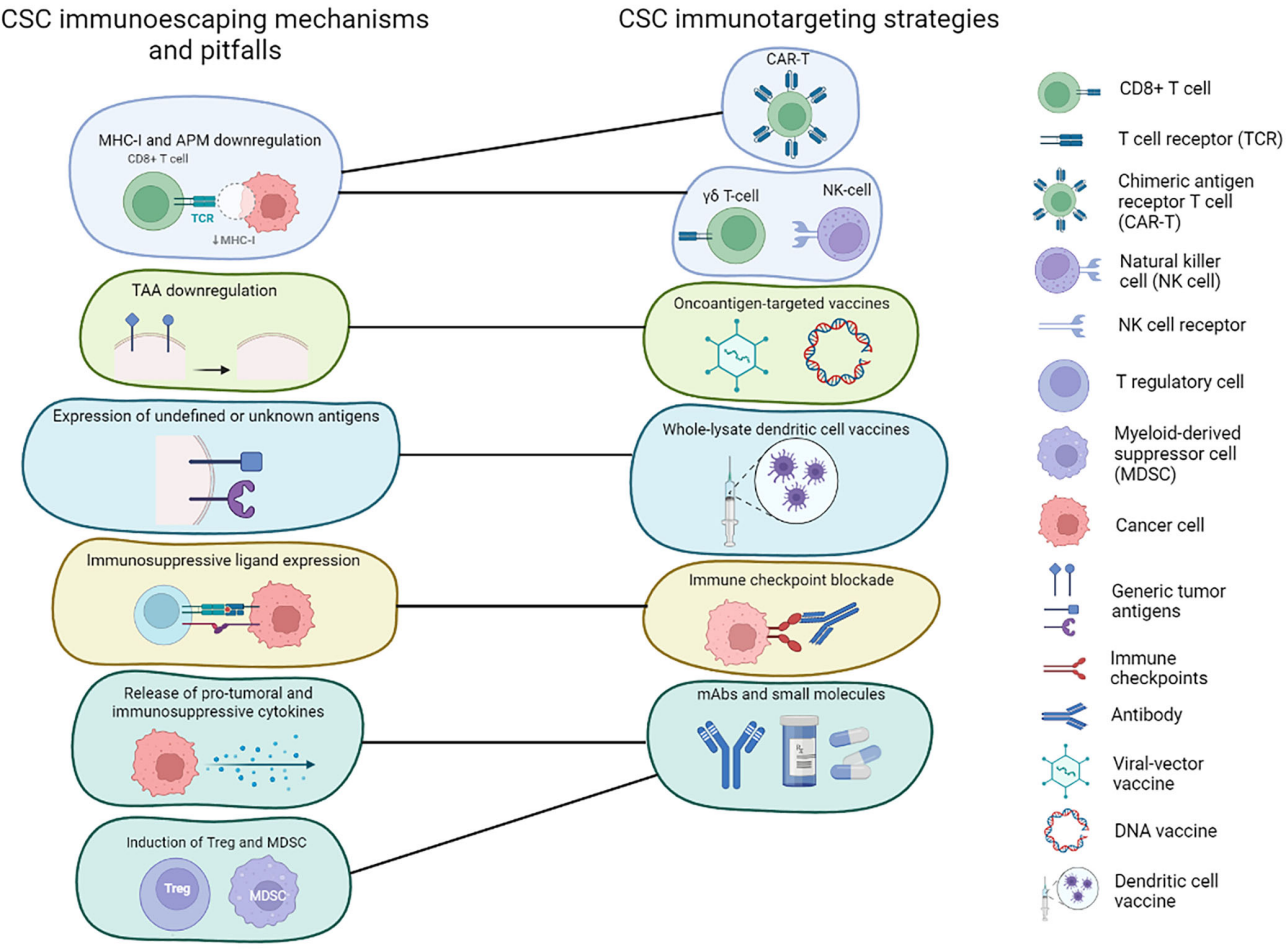
Immunotherapies targeting CSCs and CSC immunoescaping mechanism. Schematic representation of the main immunoevasive mechanisms adopted by BCSCs and how these can be addressed using different immunotherapeutic strategies.

A further complication is the ability of CSCs to evade immune system recognition, which raises doubt in the applicability of some immunotherapeutic approaches to CSC targeting. Moreover, CSCs contribute to the induction of an immunosuppressive microenvironment, which is a reported cause of immunotherapy’s low efficacy in BC (91). This highlights the importance of using agents that block such immunosuppressive pathways (**Figure 1**).

It is clear that mono(immune)therapy is not an ideal solution for BCSCs. ICIs and microenvironment-modulating agents provide the opportunity to revert the immunoevasive properties of BCSCs, while vaccine formulations that are not restrained to known BCSC antigens, such as using peptides or lysates from BCSC-enriched populations to pulse autologous DC, would allow an immune response to be mounted against all BCSC subsets. Other interesting approaches include targeting membrane molecules with a role in BCSC function, the so called “oncoantigens” (92): among others, CD44, xCT, Chondroitin Sulfate Proteoglycan 4 and Cripto1 (58, 61, 71, 93) that, when inhibited, would compromise the stem-like properties of cancer cells, making them sensitive to standard therapies. This, in combination with ICIs and bulk-shrinking therapies, would be more effective than single approaches in preventing phenotype switch, de-differentiation and eventually relapse.

## Data Availability Statement

The original contributions presented in the study are included in the article/supplementary material. Further inquiries can be directed to the corresponding author. The clinical trials described in this review can be found on the website ClinicalTrials.gov (https://clinicaltrials.gov/).

## Author Contributions

RR and LC wrote the first draft of the manuscript, FC critically revised its content and form, ADL performed the clinical trial search and produced tables and figures. All authors contributed to manuscript revision, read, and approved the submitted version

## Funding

This review article was supported by grants from the Italian Association for Cancer Research to FC (grant number IG 21468) and to LC (grant number IG 25766), from the University of Turin and from the Fondazione Ricerca Molinette. RR was supported by Fondazione Umberto Veronesi.

## Conflict of Interest

The authors declare that the article was written in the absence of any commercial or financial relationships that could be construed as a potential conflict of interest.

## Publisher’s Note

All claims expressed in this article are solely those of the authors and do not necessarily represent those of their affiliated organizations, or those of the publisher, the editors and the reviewers. Any product that may be evaluated in this article, or claim that may be made by its manufacturer, is not guaranteed or endorsed by the publisher.
